# The Birthplace of Cholera

**Published:** 1892-11-05

**Authors:** 


### THE BIRTH PLACE OF CHOLERA.

From the weekly report of the Académie des Sciences at Paris we quote the following remarks of M. de Tholozan on the recurrent march of certain previous visitations of cholera. He shows by a diagram noting the exact dates at which the cholera made its appearance in the visitation of 1846-1849, that the disease advanced from Turkestan upon India, and progressed in that empire from West to East, contrary to the direction of the epidemics which come from India from endemic air, and proceed from S. E. to N. W. Twenty-two years of investigation into the geographical chronology of the cholera have convinced M. Tholozan "that the points of emergence of cholera epidemics should be considered as their birth place. The idea of making the different pandemic manifestations of cholera which have devastated Europe, come direct from India, can no longer be sustained. In Europe alone two striking examples, in 1852 and 1869, have furnished a formal contradiction to the theories which restricted the dangers of contamination to the East. The epidemic of 1852 came from the confines of Poland and Germany and took its departure thence. The epidemic of 1869-1873 repeated the same facts in Ukraine. Attempts are sometimes made to lessen the importance of these facts by pointing out that though these epidemics did not take their rise in India, they were but the revival of former epidemics which did. Now what causes a pervading epidemic or a 'pandemic,' is just this revivification of the cholera germ,—its complete revivification with all its former attributes. This is in fact equivalent to an actual germination, since even in India it is similar revivifications which perpetuate not only the annual disorder, but those epidemics which appear every three, four, or five years. Here then is the leading, primordial fact which dominates the whole history of the cholera. It is on this point that micro-biological researches must be concentrated. What difference in morphology, in virulence, or in faculty of reproduction exists between the germs of epidemics which die out on the spot, and those of epidemics which sometimes rekindle everywhere, and though not taking their rise in India, are able to invade the entire world?"

